# Supplementary material for: Identification of photoperiod-induced specific miRNAs in the adrenal glands of Sunite sheep (Ovis aries)
Source: Front Vet Sci. 2022 Jul 22;9:888207. doi: 10.3389/fvets.2022.888207 (PMC9354845; doi:10.3389/fvets.2022.888207)
Supplement: Supplementary file 1 [file Data_Sheet_1.ZIP › Supplementary file/miRDeep2 file.zip!/miRDeep2 file/Know_Structure/SP42A1/oar-mir-30a.pdf]

miRBase precursor : oar-mir-30a
Total read count : 85913
oar-miR-30a-5p read count : 80450
oar-miR-30a-3p read count : 5463
remaining reads : 0

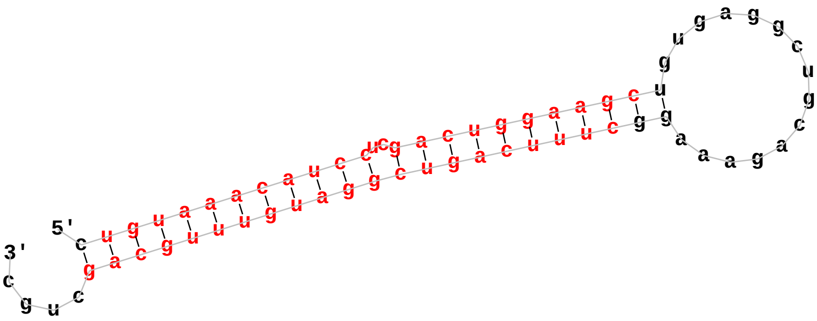

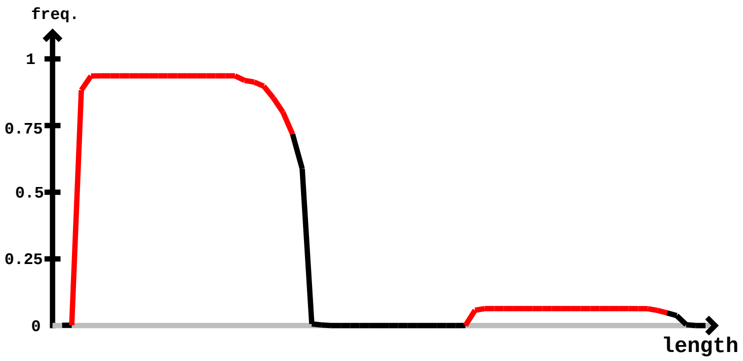


```
                                            oar-miR-30a-3p
   oar-miR-30a-5p
5'- cuguaaacauccucgacuggaagcugugaggcugcagaaaggcuuucagucggauguuugcagcugc   -3'    exp
    ((((((((((((..(((((((((((...............)))))))))))))))))))))))....    reads      mm         sample
    cuguaaacauccucgacu.................................................    1          0          seq
    cuguaaacauccuUgacu.................................................    1          1          seq
    cuguaaacauccucgacugg...............................................    4          0          seq
    cuguaaacauccucgacugga..............................................    11         0          seq
    cuguaaacauccuUgacugga..............................................    2          1          seq
    cuguaaacauccuUgacuggaa.............................................    2          1          seq
    cuguaaacauccucgacuggaa.............................................    13         0          seq
    cuguaaacauccucgacuggaag............................................    6          0          seq
    cuguaaacauccuUgacuggaag............................................    2          1          seq
    cuguaaacauccucgacuggaagc...........................................    24         0          seq
    cuguaaacauccuUgacuggaagc...........................................    3          1          seq
    cuguaaacauccuUgacuggaagcu..........................................    15         1          seq
    cuguaaacauccucgacuggaagcu..........................................    20         0          seq
    .uguaaacauccucgacu.................................................    180        0          seq
    .uguaaacauccuUgacu.................................................    1291       1          seq
    .uguaaacauccucgacug................................................    140        0          seq
    .uguaaacauccuUgacug................................................    392        1          seq
    .uguaaacauccuUgacugg...............................................    707        1          seq
    .uguaaacauccucgacugg...............................................    618        0          seq
    .uguaaacauccucgacugga..............................................    3103       0          seq
    .uguaaacauccuUgacugga..............................................    676        1          seq
    .uguaaacauccuUgacuggaa.............................................    790        1          seq
    .uguaaacauccucgacuggaa.............................................    3785       0          seq
    .uguaaacauccucgacuggaag............................................    4636       0          seq
    .uguaaacauccuUgacuggaag............................................    2372       1          seq
    .uguaaacauccuUgacuggaagc...........................................    1883       1          seq
    .uguaaacauccucgacuggaagc...........................................    9154       0          seq
    .uguaaacauccuUgacuggaagcu..........................................    28249      1          seq
    .uguaaacauccucgacuggaagcu..........................................    17642      0          seq
    .uguaaacauccuUgacuggaagcug.........................................    60         1          seq
    .uguaaacauccucgacuggaagcug.........................................    18         0          seq
    .uguaaacauccucgacuggaagcugu........................................    8          0          seq
    .uguaaacauccuUgacuggaagcugu........................................    34         1          seq
    ..guaaacauccuUgacugg...............................................    4          1          seq
```

```
                                          oar-miR-30a-3p
 oar-miR-30a-5p
cuguaaacauccucgacuggaagcugugaggcugcagaaaggcuuucagucggauguuugcagcugc

..guaaacauccuUgacugga..............................................    10     1     seq
..guaaacauccuUgacuggaa.............................................    3      1     seq
..guaaacauccucgacuggaa.............................................    7      0     seq
..guaaacauccucgacuggaag............................................    11     0     seq
..guaaacauccuUgacuggaag............................................    27     1     seq
..guaaacauccucgacuggaagc...........................................    23     0     seq
..guaaacauccuUgacuggaagc...........................................    41     1     seq
..guaaacauccucgacuggaagcu..........................................    160    0     seq
..guaaacauccuUgacuggaagcu..........................................    3867   1     seq
..guaaacauccucgacuggaagcug.........................................    3      0     seq
..guaaacauccuUgacuggaagcug.........................................    243    1     seq
..guaaacauccuUgacuggaagcugu........................................    148    1     seq
..guaaacauccucgacuggaagcugu........................................    1      0     seq
...uaaacauccucgacugga..............................................    1      0     seq
...uaaacauccucgacuggaag............................................    1      0     seq
...uaaacauccucgacuggaagc...........................................    1      0     seq
...uaaacauccuUgacuggaagc...........................................    1      1     seq
...uaaacauccuUgacuggaagcu..........................................    24     1     seq
...uaaacauccucgacuggaagcu..........................................    16     0     seq
...uaaacauccuUgacuggaagcug.........................................    7      1     seq
...uaaacauccuUgacuggaagcugu........................................    7      1     seq
.....aacauccuUgacuggaagcugu........................................    1      1     seq
......acauccuUgacuggaagcu..........................................    1      1     seq
.........................................gcuuucagucggauguuuAc......    4      1     seq
.........................................gcuuucagucggauguuuAca.....    9      1     seq
.........................................gcuuucagucggauguuuAcag....    9      1     seq
.........................................gcuuucagucggauguuuAcagc...    4      1     seq
..........................................cuuucagucggauguuu........    13     0     seq
..........................................cuuucagucggauguuug.......    2      0     seq
..........................................cuuucagucggauguuuA.......    16     1     seq
..........................................cuuucagucggauguuuAc......    426    1     seq
..........................................cuuucagucggauguuugc......    80     0     seq
..........................................cuuucagucggauguuugca.....    72     0     seq
..........................................cuuucagucggauguuuAca.....    709    1     seq
..........................................cuuucagucggauguuuAcag....    614    1     seq
..........................................cuuucagucggauguuugcag....    205    0     seq
..........................................cuuucagucggauguuuAcagc...    1911   1     seq
..........................................cuuucagucggauguuugcagc...    779    0     seq
..........................................cuuucagucggauguuugcagcu..    82     0     seq
...........................................uuucagucggauguuug.......    1      0     seq
...........................................uuucagucggauguuuAc......    28     1     seq
...........................................uuucagucggauguuugc......    12     0     seq
...........................................uuucagucggauguuuAca.....    13     1     seq
...........................................uuucagucggauguuugca.....    1      0     seq
...........................................uuucagucggauguuuAcag....    19     1     seq
...........................................uuucagucggauguuugcag....    13     0     seq
...........................................uuucagucggauguuuAcagc...    134    1     seq
...........................................uuucagucggauguuugcagc...    103    0     seq
...........................................uuucagucggauguuugcagcu..    200    0     seq
...........................................uuucagucggauguuugcagcug.    2      0     seq
............................................uucagucggauguuugcagc...    2      0     seq
```
